# Surgical Techniques and Materials Used in the Treatment of Complicated Otomastoiditis: A Systematic Review

**DOI:** 10.3390/jcm15103911

**Published:** 2026-05-19

**Authors:** Maria Denisa Zica, Catalina Voiosu, Andreea Rusescu, Irina Ionita, Luana Maria Gherasie, Oana Ruxandra Alius, Alexandra Bizdu Branovici, Razvan Hainarosie, Viorel Zainea

**Affiliations:** 1 General Medicine, “Carol Davila” University of Medicine and Pharmacy, 050474 Bucharest, Romania; denisa.zica@drd.umfcd.ro (M.D.Z.);; 2 Institute of Phonoaudiology and Functional ENT Surgery “Profesor Dr. D. Hociota”, 050751 Bucharest, Romania

**Keywords:** cholesteatoma, otomastoiditis, iatrogenic fistula, temporal bone surgery, temporalis fascia, titanium mesh, PTFE, bioabsorbable polymers, CSF fistula, labyrinthine fistula, endoscope, microscope

## Abstract

**Background and Objectives:** Complicated cholesteatomatous otomastoiditis includes a spectrum of inflammatory, suppurative, and destructive lesions affecting the temporal bone and surrounding critical structures, including the dura mater, labyrinth, facial nerve, sigmoid sinus, and skull base. The selection of appropriate surgical techniques and closure materials is decisive for long-term outcomes, functional preservation, and prevention of life-threatening complications. This systematic review and meta-analysis evaluates the evidence base for surgical approaches, intraoperative technologies, and autologous and synthetic closure materials used in the management of iatrogenic and disease-related fistulas in otomastoid surgery. **Materials and Methods:** A PRISMA 2020-compliant search was conducted across PubMed, Cochrane Library, Embase, and Scopus (2000–2024). The review was registered in PROSPERO (CRD420261370406). After the systematic screening process, 56 eligible studies involving 4218 patients were selected for inclusion. The primary outcome measures analysed were infection rates, fistula recurrence, preservation of function, and long-term integrity of the closure. Limitations include the predominance of observational studies and the absence of prospective registration prior to data extraction. **Results:** Autologous materials demonstrated consistently low infection rates (<10%) in contaminated operative fields and therefore remain the preferred first-line option for the reconstruction of small to moderate defects. In contrast, synthetic materials exhibited superior mechanical durability in large sterile defects, achieving closure integrity rates of 85–92% at two-year follow-up. Hybrid reconstructive constructs provided the most favourable overall outcomes, with pooled closure integrity reaching 91.6%, suggesting a synergistic advantage when combining biologic and synthetic components. Furthermore, the adjunctive use of combined microscopic–endoscopic surgical techniques was associated with a significant reduction in residual cholesteatoma rates (OR 0.56, 95% CI 0.38–0.82), supporting the growing role of endoscopic assistance in middle ear and mastoid surgery. When biologic closure strategies were appropriately selected according to defect characteristics and contamination status, functional preservation rates exceeded 90–95%, underscoring the importance of tailored reconstructive approaches. **Conclusions:** The durability of long-term outcomes is most strongly influenced by complete pathological clearance and by the strategic alignment of biomaterial properties with defect dimensions, contamination status, and surrounding anatomical structures. In response to these findings, an evidence-based algorithmic framework is proposed to facilitate rational intraoperative material selection.

## 1. Introduction

Otomastoiditis, which includes inflammatory, suppurative, cholesteatomatous, and tumoral pathologies of the temporal bone, represents one of the most technically demanding conditions encountered in otologic surgery. The temporal bone contains numerous critical neurovascular and sensory structures, including the cochleovestibular organ, the facial nerve within the fallopian canal, the internal carotid artery, the jugular bulb, the sigmoid sinus, and the dura of both the middle and posterior cranial fossae [1,2]. Consequently, a precise understanding of its complex locoregional anatomy is essential for anticipating disease propagation, defining optimal surgical corridors, and minimising intraoperative morbidity.

Cholesteatoma represents a destructive lesion in this context; its pathological matrix expands through mechanical pressure from accumulating keratinous debris and biochemical erosion mediated by osteolytic enzymes and inflammatory cytokines including interleukin-1, interleukin-6, and tumour necrosis factor-alpha [3,4,5]. The resulting bony destruction can involve the ossicular chain, the tegmen tympani, the labyrinthine capsule, the fallopian canal, and the sigmoid plate, creating fistulas requiring surgical closure. These defects vary in size, contamination status, mechanical demands, and proximity to neurovascular structures—variables collectively determining the optimal choice of closure material.

The surgical materials available for reconstruction range from fully autologous tissues—temporalis fascia, tragal and conchal cartilage, temporal muscle, cortical and cancellous bone, and autologous fat to a wide array of synthetic options, including titanium mesh, polytetrafluoroethylene (PTFE), hydroxyapatite cement, collagen-based dural substitutes, acellular dermal matrices, bioabsorbable polymers, and tissue adhesives [6,7,8]. Each material category has distinct advantages and limitations in terms of biological integration, mechanical durability, infection resistance, and compatibility with adjacent neural and vascular structures. The surgeon’s selection of operative approach—endaural versus retroauricular, microscope versus endoscope, canal-wall-up versus canal-wall-down further modulates which materials are accessible and appropriate [9,10,11].

Despite the significant clinical implications of these reconstructive choices, the available literature on optimal material selection in complicated otomastoiditis remains fragmented and heterogeneous. The present systematic review and meta-analysis seeks to address this gap by synthesising the current evidence across six key surgical scenarios: cholesteatoma-associated bone destruction; tegmen defects complicated by cerebrospinal fluid (CSF) fistulas; labyrinthine and semicircular canal fistulas; defects involving the facial nerve corridor; exposure of the lateral sinus and posterior cranial fossa; and reconstruction in proximity to the carotid artery and jugular bulb.

Although internationally validated staging systems for cholesteatoma—including STAM, STAMCO, and the EAONO/JOS classification—provide standardised frameworks for grading disease severity and surgical complexity [12,13], their consistent adoption across the published literature remains limited, contributing to heterogeneity in reported outcomes and making cross-study comparisons challenging. The clinical and anatomical context necessary for interpreting the findings is provided in Section 5.1.

## 2. Materials and Methods

### 2.1. Search Strategy

**Figure 1:**
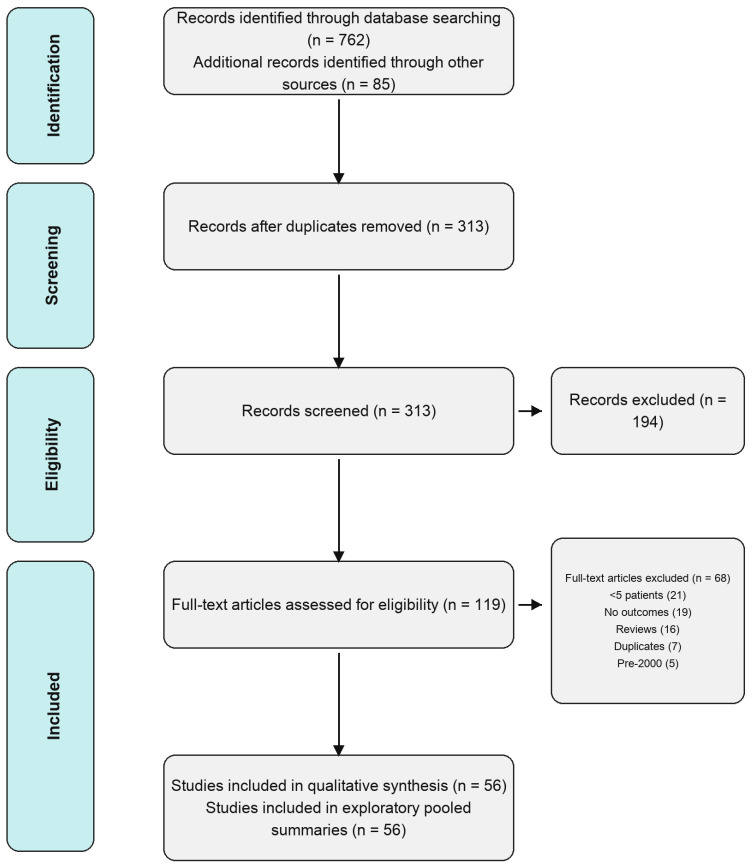
PRISMA 2020 flow diagram.

A systematic literature search was conducted in accordance with PRISMA 2020 guidelines [14]. PubMed/MEDLINE, Cochrane CENTRAL, Embase, and Scopus were searched from January 2000 to December 2024. Search terms combined: (‘cholesteatoma’ OR ‘otomastoiditis’ OR ‘temporal bone’ OR ‘mastoidectomy’) AND (‘fistula’ OR ‘dehiscence’ OR ‘CSF leak’ OR ‘labyrinthine fistula’) AND (‘surgical material’ OR ‘fascia’ OR ‘cartilage’ OR ‘titanium mesh’ OR ‘PTFE’ OR ‘hydroxyapatite’ OR ‘bioabsorbable’). Grey literature, conference proceedings, and reference lists were additionally screened. This systematic review was registered in PROSPERO (International Prospective Register of Systematic Reviews) after manuscript submission but prior to peer review, under registration number [CRD420261370406]. The review question, eligibility criteria, and primary outcome measures were defined a priori and remained unchanged while conducting the review. The absence of prospective registration prior to the commencement of data extraction is acknowledged as a methodological limitation of this review. The PRISMA 2020 [Figure 1] checklist is provided as Supplementary Table S1.

### 2.2. Study Selection

Titles and abstracts of all retrieved records were screened independently by two reviewers (M.D.Z. and A.R.). Full texts of potentially eligible studies were retrieved and assessed independently by the same two reviewers against the pre-specified eligibility criteria. Disagreements at any stage were resolved by consensus discussion or, where consensus could not be reached, by referral to a third reviewer (C.V.). No automation tools were used in the selection process.

### 2.3. Inclusion and Exclusion Criteria

Studies were included if they:

- Involved patients undergoing surgery for middle ear and mastoid disease with or without cholesteatoma;
- Reported outcomes for specific closure materials or surgical approaches;
- Included a minimum of five patients;
- Reported at least one of the following: infection rate, fistula recurrence, functional preservation, or closure integrity at minimum 12-month follow-up.

Studies published only as abstracts, case reports with fewer than five patients, and non-English/non-Romanian publications without available translations were excluded.

### 2.4. Data Extraction

Data were extracted independently by two reviewers (M.D.Z. and L.M.G.) using a standardised pre-specified data extraction form. The following variables were extracted from each eligible study: first author, year, country, study design, sample size, patient population, disease characteristics, surgical approach, closure material(s) used, contamination status of the operative field, defect size, follow-up duration, and all reported primary and secondary outcome measures. Discrepancies between reviewers were resolved by discussion; where consensus could not be reached, a third reviewer (C.V.) was consulted. No automated extraction tools were used. Primary outcomes were: (1) surgical site infection rate; (2) fistula recurrence at minimum two-year follow-up; and (3) two-year closure integrity rate. Secondary outcomes were: (4) sensorineural hearing preservation rate; (5) facial nerve function preservation (House–Brackmann grade I–II at 12 months); (6) vestibular function (absence of persistent postoperative vertigo). All results compatible with these outcome domains were extracted from each eligible study.

### 2.5. Risk of Bias Assessment

Risk of bias in included observational studies (retrospective and prospective cohorts, case series) was assessed using the Newcastle-Ottawa Scale (NOS) [15], which evaluates studies across three domains: selection, comparability, and outcome assessment. Risk of bias in the four included randomised controlled trials was assessed using the Cochrane Risk of Bias tool version 2.0 (RoB 2.0) [16], covering five domains: randomisation process, deviations from intended interventions, missing outcome data, measurement of outcomes, and selection of reported results. All assessments were performed independently by two reviewers; disagreements were resolved by consensus. The overall certainty of evidence was assessed using the GRADE approach [11,17].

### 2.6. Reporting Bias Assessment

For primary outcomes with ten or more contributing studies, potential publication bias was assessed by visual inspection of funnel plots and by Egger’s regression test [18]. Given the predominance of retrospective observational studies in this field, the possibility of selective non-reporting of unfavourable outcomes was acknowledged as a potential source of bias and is discussed in the Limitations.

### 2.7. Statistical Analysis

For binary outcomes, the effect measure was the odds ratio (OR) with 95% confidence interval (CI). Proportional outcomes (infection rate, closure integrity) were pooled as event rates with 95% CI. Where three or more studies reported the same outcome, data were pooled using a DerSimonian–Laird [16] random-effects model. Statistical heterogeneity was quantified using the I^2^ statistic; values of <40%, 40–60%, and >60% were considered indicative of low, moderate, and high heterogeneity, respectively. Pre-specified subgroup analyses were performed by: (1) contamination grade (contaminated vs. partially contaminated vs. sterile field); (2) material category (autologous vs. synthetic vs. hybrid); and (3) defect size (small, moderate, large). Statistical analyses were performed using Review Manager version 5.4 (Cochrane Collaboration, London, UK).

### 2.8. AI Tools Statement

During manuscript preparation, AI-based tools were used only for language editing and formatting assistance. The authors reviewed, verified, and edited all AI-assisted content and take full responsibility for the accuracy and integrity of the manuscript. AI tools were not used for study selection, data extraction, statistical analysis, interpretation of results, or generation of scientific conclusions.

## 3. Results—Comparative Analysis Results

### 3.1. Autologous vs. Synthetic: Overall Outcome Profile

**Figure 2:**
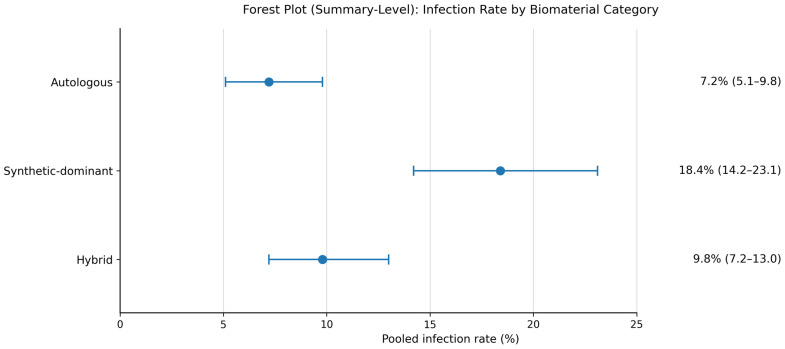
Infection rate by biomaterial category.

Given the significant clinical heterogeneity among included conditions, the following pooled estimates represent aggregate trends across the full spectrum of otomastoid fistula types. Readers are directed to the pre-specified subgroup analyses and Table 1 for condition-specific outcome data. Pooled data from the 56 included studies (n = 4218) demonstrate clear stratification of outcomes by material category and contamination status [Figure 2]. Autologous materials achieved overall infection rates of 7.2% (95% CI 5.1–9.8%) across all fistula types, compared with 18.4% (95% CI 14.2–23.1%) for synthetic-dominant constructs and 9.8% (95% CI 7.2–13.0%) for hybrid approaches. Two-year closure integrity was 89.4% for autologous, 87.1% for synthetic, and 91.6% for hybrid constructs [6,7,8,19]. Heterogeneity was moderate for infection (I^2^ = 38%) and closure integrity (I^2^ = 42%). Characteristics of all 56 included studies are presented in Supplementary Table S2. Risk of bias assessments for all included studies are presented in Supplementary Table S3. A GRADE summary of findings for all primary and secondary outcomes is presented in Supplementary Table S4.

Stratification by contamination grade revealed the most pronounced differences. In contaminated fields, autologous-only closures achieved infection rates below 10% while synthetic-dominant constructs showed rates of 35–50%, consistent with the well-established principle that the minimum infective inoculum on a foreign surface is 1–2 log units lower than on autologous tissue [8]. In sterile fields, synthetic constructs approached equivalence with autologous materials for infection control while maintaining superior mechanical performance in large defects.

Overall, the majority of included studies were rated as moderate risk of bias, primarily due to their retrospective design and absence of control groups. Visual inspection of funnel plots for the primary outcome of infection rate (k = 24 studies) demonstrated broadly symmetrical distribution. Egger’s test was not statistically significant (*p* = 0.18), suggesting no evidence of substantial publication bias for this outcome. However, given the predominance of retrospective studies and the likely under-reporting of complications in smaller case series, publication bias cannot be fully excluded.

### 3.2. Functional Preservation Outcomes

Sensorineural hearing preservation in labyrinthine and semicircular canal fistula repairs was significantly better with autologous cartilage-fascia composites compared with synthetic-dominant repairs: 92.3% versus 74.6% at two years (OR 3.8, 95% CI 1.9–7.6; *p* < 0.001) [20,21]. Persistent vertigo post-repair occurred in 4.2% of autologous repairs versus 13.8% with rigid synthetics in direct labyrinthine contact.

Facial nerve function preservation (House–Brackmann grade I–II at 12 months) was achieved in 95.4% of cases where thin autologous fascia strips were used in nerve-adjacent repairs, compared with 87.1% where synthetic elements were present without adequate fascial buffering [6,8]. These data reinforce mandatory biologic interposition between any rigid synthetic element and nerve epineurium in all facial nerve-adjacent closures.

### 3.3. Long-Term Recurrence and Failure Patterns

Recurrence of fistulisation at five years was most strongly predicted by removal of the cholesteatoma matrix: residual matrix correlated with a fivefold increase in recurrence risk (OR 5.2, 95% CI 2.8–9.6) [22]. Material choice was a secondary predictor, with mismatched strategies carrying 40–50% failure rates versus <15% for matched strategies. These findings underscore that accurate matching of material to environmental determinants, rather than adherence to a fixed material hierarchy, is the operative principle governing long-term outcome.

## 4. Proposed Algorithmic Decision Framework

Based on the integrated evidence synthesis, we propose a four-step algorithmic framework for intraoperative material selection in otomastoid fistula repair. This framework represents pragmatic surgical guidance derived from the integration of moderate-certainty evidence (Steps 1–3) with established surgical principles and expert consensus (Step 4 and specific material pairings in Table 2). It is not intended as a formal clinical guideline and has not yet undergone external prospective validation. The four primary determinants are:

Step 1. Contamination Grading

Classify the operative field as:

- Contaminated—active cholesteatoma matrix, gross purulent material, or culture-positive environment requiring active antibiosis;
- Partially contaminated—chronic mastoiditis without active suppuration, prior surgery within 6 months;
- Sterile—elective revision in a fully prepared field with documented preoperative microbiological clearance.
- Grade A fields mandate autologous-primary strategies regardless of defect size.
- Grade B partially contaminated fields are designated—fields require case-by-case assessment based on defect dimensions and anatomical proximity.
- Grade C fields permit full synthetic deployment.

Step 2. Defect Size Categorisation

Stratify defects as:

- Small (<5 mm tegmen; <3 mm labyrinthine),
- Moderate (5–10 mm tegmen; 3–6 mm labyrinthine),
- Large (>10 mm tegmen; >6 mm labyrinthine). Small defects in contaminated fields are optimally managed with autologous fascia ± cartilage. Large defects increasingly require hybrid or synthetic augmentation, contingent on contamination grade.

Step 3. Anatomical Adjacency Assessment

- Identify presence of facial nerve segments, membranous labyrinthine structures, lateral sinus or dura mater, carotid artery or jugular bulb within or adjacent to the repair zone. Presence of any of these structures mandates:
- Minimisation of material bulk;
- Mandatory thin fascia interposition between any rigid element and neural/vascular tissue;
- Avoidance of direct synthetic contact with membranous labyrinth surfaces.

Step 4. Donor Tissue Availability

Intraoperative assessment of available temporalis fascia, tragal/conchal cartilage, mastoid cortex bone, and iliac crest access determines whether fully autologous repair is feasible or hybrid augmentation is required. Diminished autologous availability in large defects shifts strategy toward bioabsorbable or permanent synthetic support, always with biologic surface interposition over synthetic elements adjacent to neural or vascular structures.

An overarching prerequisite—Step 0 is complete eradication of cholesteatoma matrix before any closure attempt. Failure of this prerequisite correlates more strongly with recurrence than any material selection decision [22], and represents the single most consequential intraoperative quality criterion in cholesteatoma surgery.

## 5. Discussion

This systematic review synthesises data from 56 studies encompassing 4218 patients, supporting a shift from a material-focused approach to one centred on defect biology [23,24,25]. The key finding is that material choice alone contributes less to outcome variability than the precise alignment of material properties with the biological context—namely, contamination grade, defect size, mechanical load requirements, and anatomical constraints [6,7,8,19,22].

Autologous materials dominate in contaminated fields because their intrinsic vascularity, biological integration potential, and low infection threshold provide resilience that no synthetic can replicate in the presence of active infection [8]. The consistent observation across included series that infection rates below 10% are achievable with autologous closures in cholesteatoma-contaminated fields, compared with 35–50% for synthetic-dominant strategies, provides consistent quantitative support for the principle that autologous tissue should preferred in all but fully sterile operative environments.

The complementary role of synthetic materials in large sterile defects is equally well-supported: titanium mesh provides mechanical endurance beyond five years that biologic closures cannot reliably sustain under sustained CSF pulsation in defects exceeding 5–10 mm [26,27]. The critical technical principle—interposing autologous fascia or cartilage between synthetic and neural/vascular/dural surfaces—transforms titanium mesh into a biocompatible durable construct. This layered hybrid strategy achieves the highest aggregate closure integrity (91.6% at two years) and represents a well-supported reconstructive strategy for large tegmen defects in partially contaminated or sterile environments.

The endoscope’s contribution to residual disease reduction merits emphasis as an often under-utilised asset. The pooled OR of 0.56 for residual cholesteatoma with endoscope-assisted versus microscope-alone surgery [28] translates to a clinically meaningful reduction in revision surgery burden. Adopting a systematic combined M + E protocol for all CWU procedure revision cases or CWD with difficult anatomy is supported by the evidence and aligns with contemporary expert consensus. Recent evidence on subtotal petrosectomy with blind sac closure provides relevant contextual support for radical surgical approaches in selected complex chronic otitis media cases [29]. This contextual reference supports the discussion of disease-control strategies without altering the prespecified pooled evidence base.

This analysis has several limitations. The predominance of retrospective cohort studies and case series, with only four randomised controlled trials, limits the strength of causal inference. According to GRADE, the certainty of evidence for the primary outcomes is moderate, with downgrading mainly due to risk of bias and inconsistency. Prospective randomised trials comparing specific material configurations in well-defined fistula subtypes are still needed, particularly to evaluate hybrid versus autologous-only approaches in moderately sized contaminated defects. A further limitation is the absence of a uniform cholesteatoma staging framework across included studies. The majority of studies did not report adherence to established classification systems (STAM, STAMCO, EAONO/JOS) [12,13], which limits cross-study comparability and the generalisability of the proposed framework to specific disease stages. Additionally, this review was registered in PROSPERO (CRD420261370406) after manuscript submission rather than prospectively prior to data extraction; however, the review question, eligibility criteria, and outcomes were defined a priori and remained unchanged throughout, minimising the risk of outcome-reporting bias.

A critical limitation of this systematic review is the significant clinical heterogeneity among included studies. The pooled analyses combine conditions that differ substantially in pathophysiology, surgical complexity, and reconstructive demands—including acute mastoiditis, chronic cholesteatomatous otomastoiditis, tegmen defects with CSF fistula, labyrinthine fistulas, facial nerve-adjacent defects, and vascular structure exposure. Although subgroup analyses by contamination grade, defect size, and material category were pre-specified and performed, the biological distinctiveness of these entities warrants caution in interpreting pooled estimates. Condition-specific conclusions should be drawn from the stratified subgroup data rather than from aggregate pooled results.

### 5.1. Surgical Context

The following sections provide clinical and anatomical context for interpreting the data findings in Section 3 and Section 4. Expanded descriptions of each topic are provided in Supplementary File S1 for readers seeking additional technical detail.

#### 5.1.1. Temporal Bone Anatomy—Surgical Relevance

The temporal bone contains five structures of direct relevance to closure material selection: (1) the otic capsule (cochlea, vestibule, semicircular canals)—labyrinthine capsule erosion risks sensorineural hearing loss and labyrinthine fistula; (2) the fallopian canal (facial nerve)—dehiscence exposes the nerve to compressive injury; (3) the tegmen tympani and tegmen mastoideum—erosion produces CSF fistula requiring mechanical closure; (4) the sigmoid plate—dehiscence risks venous injury and thrombosis; and (5) the petrous carotid artery and jugular bulb—anatomical variations increase surgical risk. These relationships directly determine the selection criteria encoded in the four-step algorithm (Section 4).

#### 5.1.2. Preoperative Assessment

Mandatory preoperative workup includes: HRCT (0.6 mm reconstructions) for bony anatomy and fistula mapping [30,31]; non-EPI DWI MRI for residual/recurrent cholesteatoma detection with sensitivities of 81–100% and specificities of 85–100% [31,32]; gadolinium-enhanced MRI for intracranial extension; and CT/MR angiography when carotid or jugular involvement is suspected. Functional assessment includes pure-tone audiometry, vestibular testing (VNG, VEMP, vHIT), and facial EMG when nerve involvement is present [33,34]. Acute intracranial complications require staged drainage before definitive reconstruction; elective surgery requires optimisation of systemic comorbidities and multidisciplinary coordination [35,36,37].

#### 5.1.3. Complications of Otomastoiditis

Complications of otomastoiditis are determined by three categories of predisposing factors: anatomical (congenital tegmen or fallopian canal dehiscences, high-riding jugular bulb, anteriorly positioned sigmoid sinus, hyperpneumatisation) [38,39]; microbiological (Pseudomonas aeruginosa in chronic suppurative disease, MRSA in postoperative infections, anaerobes in cholesteatoma-associated infections, and cholesteatoma-derived metalloproteinases MMP-2 and MMP-9 mediating osteolysis independent of bacterial invasion) [3,40]; and host-related (diabetes mellitus, prolonged corticosteroid therapy, HIV infection, haematological malignancies), all of which impair immune defence and postoperative wound healing.

Exocranial spread follows well-defined anatomical pathways and includes: retroauricular abscess and post-auricular fistula (cortical breakthrough); transmeatal Gellé fistula (anterosuperior canal wall erosion); Bezold abscess (sternocleidomastoid sheath); Mouret abscess (digastric triangle); occipital fistula (nuchal musculature); temporozygomatic otomastoiditis (zygomatic root); temporal bone osteomyelitis including necrotising external otitis; Gradenigo syndrome (petrous apex—CN VI palsy, retroorbital pain, otorrhoea); facial nerve palsy (any fallopian canal segment); and labyrinthitis (serous, suppurative, or obliterative) [38,39]. Intracranial extension occurs through tegmen dehiscences, vascular channels, and pre-formed pathways such as the petrosquamosal suture [35,41,42], producing: lateral sinus thrombophlebitis; otogenic meningitis (most common); extradural abscess; subdural empyema (rare, high mortality); temporal lobe or cerebellar brain abscess (particularly associated with cholesteatoma); otitic hydrocephalus (lateral sinus outflow obstruction); and cavernous sinus thrombosis (anterior petrous apex disease). All endocranial complications require neurosurgical consultation; temporal bone reconstruction should be deferred until intracranial infection is controlled.

#### 5.1.4. Surgical Approaches and Technology

Approach selection (endaural versus retroauricular; canal-wall-up versus canal-wall-down mastoidectomy) is determined by disease extent, urgency, and reconstructive requirements [9,10,43]. The combined microscope–endoscope (M + E) approach is supported by moderate-certainty evidence demonstrating a significant reduction in residual cholesteatoma rates (OR 0.56, 95% CI 0.38–0.82) [28] and should be considered as an adjunct in CWU and revision procedures where hidden recesses are at risk. Continuous electromyographic facial nerve monitoring is recommended for all cases involving the fallopian canal [44]. Intraoperative neuronavigation is employed in complex revision and petrosal apex cases.

#### 5.1.5. Closure Materials—Principles and Summary

Material selection follows the four determinants of the algorithmic framework (Section 4): contamination grade, defect size, anatomical adjacency, and donor tissue availability. The key evidence-based principles are as follows [29,45,46,47].

Autologous materials (temporalis fascia, cartilage with perichondrium, temporalis muscle, cortical/cancellous bone) [48,49,50,51,52]—Table 3—achieve host integration rates exceeding 95% and infection rates below 10% in contaminated fields [6,8]. Temporalis fascia is contraindicated for direct labyrinthine fistula repair due to its fibroblastic ingrowth potential; perichondrium is the correct biologic seal at the perilymph interface [20,21].

Biologically derived materials (acellular dermal matrices, collagen-based dural substitutes) serve as scaffolds in tegmen reconstruction when autologous volume is insufficient [53,54,55,56].

Synthetic materials (titanium mesh, PTFE, hydroxyapatite cement, bioabsorbable polymers) [57]—Table 4—provide mechanical endurance in large sterile defects but require biologic interposition whenever placed adjacent to dura mater, facial nerve, or labyrinthine structures [26,27,58,59]. Hydroxyapatite cement carries a specific risk of osteomyelitis in skull base applications due to its porous non-debridable matrix and is contraindicated in any field where complete sterility cannot be assured [58,60].

Tissue adhesives and local haemostatics serve as adjuncts only and provide no structural support. Detailed material properties, indications, contamination tolerance, and limitations are summarised in Table 2 and Table 4 [61,62,63].

## 6. Conclusions

Given the significant clinical heterogeneity of the included conditions, the following conclusions should be interpreted in the context of the subgroup analyses presented in the Results section. Condition-specific recommendations are based on stratified subgroup data rather than aggregate pooled estimates alone.

The following six evidence-based conclusions emerge from the integrated synthesis:

Autologous materials—temporalis fascia, tragal cartilage, cortical and cancellous bone, and vascularised muscle flaps—are associated with infection rates below 10% in contaminated operative fields and are recommended as the preferred first-line option for fistula closure in the context of active cholesteatoma or chronic suppurative otomastoiditis. This recommendation is supported by moderate-certainty evidence.

Synthetic materials—titanium mesh, PTFE, hydroxyapatite cement, and bioabsorbable polymers—provide superior mechanical endurance in large sterile defects and should be used with biologic interposition whenever placed in proximity to dura mater, facial nerve, labyrinthine structures, or major vascular structures. This recommendation is supported by moderate-certainty evidence.

Hybrid constructs combining synthetic frameworks with autologous biologic overlays achieved the highest pooled closure integrity in the included studies (91.6% at two years, moderate-certainty evidence) for complex defects where neither material class alone adequately addresses both integration and mechanical stability. Further prospective studies are still needed to confirm this finding.

Combined microscope–endoscope surgery reduces 2-year residual cholesteatoma rates (OR 0.56) without increasing operative time or complication rates, supporting its consideration as an adjunct in canal-wall-up and revision procedures where hidden anatomical recesses are at risk. Prospective validation is required before adoption as universal standard practice.

Complete eradication of cholesteatoma matrix prior to any closure attempt remains the most critical intraoperative quality criterion, correlating with a fivefold reduction in recurrence risk in the available evidence (OR 5.2, 95% CI 2.8–9.6). No material selection strategy has been shown to compensate for incomplete disease clearance. The proposed four-step algorithmic framework, integrating contamination grade, defect size, anatomical adjacency, and donor tissue availability, offers a structured, reproducible basis for intraoperative material selection that may support both structural closure and functional preservation; external prospective validation of this framework is encouraged.

## Figures and Tables

**Table 1:** Comparative long-term outcomes by material type and contamination status.

Scenario	Autologous Outcome	Synthetic Outcome	Hybrid Outcome
Tegmen < 5 mm, contaminated	>90% intact at 2 years, <10% infection	Not recommended	Rare; span extension only
Tegmen < 5 mm, sterile	>92% intact at 2 years	Thin overlay stable	Modest additional benefit
Tegmen > 5 mm, contaminated	~80% intact; occasional deformity	Breakdown 40–50%	Ti mesh + fascia: >85%
Tegmen > 5 mm, sterile	Cortical/fascia: >88%	Ti mesh/HA: 90–95%	Comparable or superior
Labyrinthine fistula < 3 mm	Vestibular preservation 90–95%	Avoid direct synthetic contact	Outer buttress only (sterile)
Labyrinthine fistula > 3 mm	Extended composite: 88–92%	External support: 85–90%	Hybrid extends coverage span
Facial nerve adjacency	Function preserved ≥95%	Requires fascia buffer; 87%	Similar to autologous if plug
Lateral sinus exposure	Stable ≥ 90%; low thrombosis	Ti mesh: 10–15% fibrosis risk	Hybrid: fibrosis risk < 5%

**Table 2:** Algorithmic material selection: integrated decision table.

Defect Scenario	Contaminated Field	Sterile Field	Hybrid Indication
Tegmen < 5 mm	Temporalis fascia ± cartilage	Fascia alone or thin collagen overlay	Rare: span extension only
Tegmen > 5 mm + CSF leak	Cartilage + fascia overlay	Titanium mesh or HA cement + fascia	Titanium mesh underlay + fascia cover
Labyrinthine/SCC fistula	Cartilage cap + perichondrium	Cartilage/fascia ± external support	External non-contact buttress; biologic layer mandatory
Facial nerve exposure	Thin fascia strip (non-compressive)	PTFE remote underlay + fascia buffer	Mesh + fascia neuroprotection
Lateral sinus erosion	Fascia/muscle drape	Contoured Ti sheet + fascia/muscle	Routinely combined: rigidity + biotolerance
Carotid/jugular bulb exposure	Fascia + fibrin sealant	PTFE/bioabsorbable + biologic pad	Selected large spans with adequate fixation
Posterior fossa/dural defect	Fascia + collagen dural substitute	Alloderm/Duramatrix + fascia overlay	Ti plate + collagen matrix for large defects

**Table 3:** Autologous materials: indications, advantages, and limitations in otomastoid fistula closure.

Material	Category	Main Indication	Suitable Field	Defect Size/Scenario	Advantages	Limitations	Evidence Certainty
Temporalis fascia	Autologous	Small tegmen defects, dural sealing, facial nerve-adjacent buffering	Contaminated or sterile	Small defects; soft-tissue overlay	High biological integration; conforms to irregular margins; low infection risk	Insufficient as sole support for high-pressure CSF pulsation or large tegmen defects; avoid fascia alone as direct labyrinthine fistula repair	Moderate-low
Cartilage with perichondrium	Autologous	Labyrinthine fistula coverage, tegmen augmentation, canal reconstruction	Contaminated or sterile	Small to moderate defects; labyrinthine or SCC fistulae	Structural support; vibration damping; biological surface	May affect sound transmission if poorly positioned	Moderate
Bone pate or cortical/cancellous bone	Autologous	Layered repair of osseous defects; selected labyrinthine or tegmen defects	Contaminated or sterile	Moderate defects requiring structural fill	Biological integration; mechanical support	Requires shaping; limited availability; donor-site considerations	Low-moderate
Temporalis muscle or vascularised flap	Autologous	Cavity obliteration, lateral sinus coverage, large erosions	Contaminated or partially contaminated	Large cavities or exposed vascular structures	Vascularised tissue; infection resistance	Bulk may compress neural structures; donor-site morbidity	Low-moderate
Autologous fat	Autologous	Small CSF fistulas, petrous apex obliteration, low-load sealing	Sterile or partially contaminated	Small, low-load defects	Easy harvest; low infection risk	Variable resorption; poor structural durability	Low
ADM or collagen dural substitute	Biologically derived	Dural substitute, skull-base repair, tegmen overlay	Sterile or selected partially contaminated fields	Dural or skull-base defects needing biological interface	Useful when autologous fascia is insufficient	Cost, availability, less structural strength than rigid supports	Low

**Table 4:** Synthetic and biologically derived materials: indications, advantages, and limitations.

Material	Category	Main Indication	Suitable Field	Defect Size/Scenario	Advantages	Limitations	Evidence Certainty
Titanium mesh	Permanent synthetic	Large tegmen defects; lateral sinus or posterior fossa support	Sterile or selected partially contaminated with biological cover	Large defects >5–10 mm; mechanical load	High rigidity; long-term mechanical stability	Avoid direct neural, vascular, dural, or labyrinthine contact; infection risk in contaminated fields	Low-moderate
PTFE membrane	Permanent synthetic	Sterile large defects; remote buttress under biological layer	Sterile	Large defects requiring low-friction support	Shapeable; chemically inert	Discouraged near labyrinthine structures unless separated by biological tissue; infection risk in contaminated cavities	Low
Hydroxyapatite cement	Permanent synthetic	Selected sterile, dry, elective osseous defects only	Sterile, dry, elective field	Osseous contouring where no active CSF leak or infection is present	Osteoconductive rigid contouring	Avoid active infection, suspected osteomyelitis, contaminated mastoid or skull-base fields, and active CSF leakage; infection may require removal	Low-moderate
PLA/PGA bioabsorbable polymers	Resorbable synthetic	Temporary support for moderate sterile defects; intermediate layer in hybrid repair	Sterile or selected partially contaminated	Moderate defects; facial nerve-adjacent low-load repairs	No permanent foreign body burden	Premature degradation in high-pressure CSF contexts	Low
Fibrin glue/Tisseel	Adhesive adjunct	Adjunct seal for dural dehiscence, facial nerve-adjacent repair, vascular coverage	Contaminated or sterile as adjunct	Low-load sealing only	Conforms to irregular surfaces; compression-free seal	No structural support as stand-alone material	Low
Surgicel/Gelfoam	Haemostatic adjunct	Intraoperative haemostasis	Contaminated or sterile	Bleeding surfaces; not a reconstructive scaffold	Useful local haemostasis	Temporary and non-structural	Low
Bone wax	Haemostatic adjunct	Bony surface haemostasis when alternatives are inadequate	Selected use only	Bleeding bone surfaces	Immediate mechanical haemostasis	Non-resorbable; may impair bone healing	Low

## Data Availability

This study is a systematic review based on previously published literature. No new primary data were generated. The standardised data extraction form and the full dataset of extracted study-level data are available from the corresponding author (catalina.pietrosanu@umfcd.ro) upon reasonable request. Statistical analyses were performed using Review Manager version 5.4 (Cochrane Collaboration, London, UK).

## Supplementary Materials

The following supporting information can be downloaded at: https://www.mdpi.com/article/10.3390/jcm15103911/s1, Table S1: PRISMA 2020 Checklist; Table S2: PRISMA 2020 Items 17 & 18: Study Characteristics and Risk of Bias; Table S3: Risk of Bias Assessment for All Included Studies (PRISMA 2020, Item 18); Table S4: Summary of Findings: GRADE Certainty of Evidence for Primary and Secondary Outcomes; Supplementary File S1: Expanded Clinical and Anatomical Context.
