# Supplementary material for: Surgical Techniques and Materials Used in the Treatment of Complicated Otomastoiditis: A Systematic Review
Source: J Clin Med. 2026 May 19;15(10):3911. doi: 10.3390/jcm15103911 (PMC13207162; doi:10.3390/jcm15103911)
Supplement: Supplementary file 1 [file jcm-15-03911-s001.zip › jcm-4280408-Supplementary File S5.pdf]

Supplementary File S5: Expanded Clinical and Anatomical Context

This supplementary file contains the expanded clinical and anatomical material that supported Section 5.1 of the main manuscript. It is provided for readers seeking additional technical detail and has been relocated from the main text in accordance with standard systematic review reporting format.

S5.1. Temporal Bone Anatomy — Detailed Description

S5.1.1. Overview of Temporal Bone Structure

The temporal bone is a highly complex osseous structure forming part of the lateral skull base and housing critical neurovascular and sensory elements. Anatomically, it is composed of four principal components: the squamous portion (pars squamosa), the tympanic portion (pars tympanica), the mastoid portion (pars mastoidea), and the petrous portion (pars petrosa) [1,10].

The petrous portion contains the otic capsule, one of the densest bones in the human body, which encloses the cochlea, vestibule, and semicircular canals. Within this region are several essential anatomical structures, including the internal auditory canal (containing cranial nerves VII and VIII), the carotid canal transmitting the internal carotid artery, and the jugular fossa accommodating the jugular bulb. Superiorly, the tegmen tympani and tegmen mastoideum form thin osseous partitions separating the middle ear and mastoid cavities from the middle cranial fossa. Posteriorly, the sigmoid plate forms the bony boundary between the mastoid cavity and the sigmoid sinus within the posterior cranial fossa.

The facial nerve traverses the temporal bone within the fallopian canal, following a complex three-segment course: the labyrinthine segment extending from the fundus of the internal auditory canal to the geniculate ganglion; the tympanic segment running horizontally along the medial wall of the tympanic cavity; and the mastoid segment descending vertically to exit through the stylomastoid foramen [2,11]. Precise knowledge of these anatomical relationships is essential in otologic surgery. Cholesteatomatous disease frequently erodes the fallopian canal, exposing the facial nerve and increasing the risk of neuropraxia or direct neural injury during surgical dissection.

The labyrinthine capsule represents a particularly critical anatomical zone. Pathological erosion may result in labyrinthine fistulas, while inadvertent surgical injury may lead to irreversible sensorineural hearing loss and vestibular dysfunction.

S5.1.2. Pathological Anatomy and Surgical Risk

Pathological processes affecting the temporal bone significantly modify the local anatomy and increase surgical complexity. In acute mastoiditis, infection typically progresses from bacterial otitis media, leading to mucosal oedema, hyperaemia, and accumulation of purulent secretions within the mastoid air cell system [12,13]. If untreated, progressive osteoclastic activity results in erosion of mastoid and cortical bone, facilitating extracranial or intracranial spread.

Chronic mastoiditis presents a distinct pathological environment. Persistent inflammation predisposes patients to cholesteatoma development. When present, progressive expansion of the keratinising epithelial matrix leads to bone destruction through matrix metalloproteinases, collagenases, and inflammatory mediators [3,14]. The resulting osseous defects — involving the ossicular chain, tegmen tympani, labyrinthine capsule, fallopian canal, or sigmoid plate — define the reconstructive challenges encountered during surgery.

S5.1.3. Surgical Risk Zones and Operative Corridors

The facial nerve corridor, tegmen plate, sigmoid sinus/sinodural angle, labyrinthine capsule, and petrous carotid/jugular bulb represent the principal surgical risk zones in temporal bone surgery. Dehiscence of the fallopian canal is frequently encountered in cholesteatoma surgery. Thinning or dehiscence of the tegmen may

result in CSF leakage. Sigmoid plate erosion may expose the venous sinus. Labyrinthine capsule erosion produces fistulas requiring perichondrium (not fascia) for repair. Anatomical variations including high jugular bulb or dehiscent carotid canal must be identified preoperatively on HRCT.

S5.2. Preoperative Assessment — Detailed Protocol

S5.2.1. Clinical and Paraclinical Evaluation

A preoperative assessment is mandatory in patients with complicated otomastoiditis. The diagnostic workup includes a complete ENT examination with otoscopy, otoendoscopy, and otomicroscopy. Functional testing involves pure-tone and speech audiometry, tympanometry, and vestibular investigations including VNG, VEMP, and vHIT [17,18]. Preoperative electromyographic facial nerve assessment is essential in all cases with clinical evidence of facial nerve involvement.

Imaging is indispensable for surgical planning. HRCT with 0.6 mm axial and coronal reconstructions delineates bony erosion, fistula anatomy, and relationships to critical structures [19,20]. Non-EPI DWI MRI has revolutionised cholesteatoma detection, providing sensitivities of 81–100% and specificities of 85–100% for residual or recurrent disease while eliminating ionising radiation [20,21]. Gadolinium-enhanced MRI identifies dural involvement, venous sinus thrombosis, and intracranial extension. CT/MR angiography is indicated whenever carotid or jugular bulb involvement is suspected.

S5.2.2. Contraindications and Timing

In acute complicated mastoiditis with intracranial extension — meningitis, brain abscess, lateral sinus thrombophlebitis — surgical drainage takes precedence, with definitive mastoidectomy planned once infection is controlled [22,23]. Elective surgery requires optimisation of systemic comorbidities and multidisciplinary review.

Key contraindications include: uncontrolled systemic infection; severe coagulopathy; active anticoagulation without washout; elevated intracranial pressure without neurosurgical management; patient refusal; procedures exceeding the surgeon's competence.

S5.2.3. Interdisciplinary Coordination

Neurosurgical consultation is mandatory for posterior or middle cranial fossa involvement [24]. Vascular surgery standby is considered in carotid or jugular bulb erosion. Continuous electromyographic facial nerve monitoring is recommended for all cases involving the fallopian canal [25]. Neuronavigation is employed in complex revision and petrosal apex cases.

S5.3. Complications of Otomastoiditis — Detail

S5.3.1. Causes and Predisposing Factors

Complications are influenced by anatomical predispositions (congenital tegmen or fallopian canal dehiscences, high jugular bulb, anteriorly positioned sigmoid sinus, hyperpneumatisation), microbiological factors (Pseudomonas aeruginosa in chronic suppurative otitis media; MRSA in postoperative infections; anaerobes and cholesteatoma-derived metalloproteinases MMP-2 and MMP-9), and host factors (diabetes mellitus, corticosteroid therapy, HIV, haematological malignancies) [3,26,27,28].

S5.3.2. Exocranial Complications

Exocranial spread from mastoid disease follows well-defined anatomical pathways: retroauricular abscess and post-auricular fistula (cortical breakthrough); Gellé fistula (anterosuperior canal wall erosion); Bezold abscess (medial cortex breakthrough into sternocleidomastoid sheath); Mouret abscess (digastric groove extension); occipital fistula (posterior cortex erosion); temporozygomatic otomastoiditis (zygomatic root extension); osteomyelitis and necrotising external otitis; Gradenigo syndrome (petrous apex — CN VI palsy, retroorbital pain, otorrhoea); facial nerve palsy; labyrinthitis (serous, suppurative, or obliterative). See Table 5 for surgical relevance of each complication.

S5.3.3. Endocranial Complications

Intracranial extension occurs through tegmen dehiscences, vascular channels, and pre-formed pathways [22,29]. Principal endocranial complications are: lateral sinus thrombophlebitis; otogenic meningitis (most common); extradural abscess; subdural empyema (rare, high mortality); temporal lobe or cerebellar brain abscess; otitic hydrocephalus; cavernous sinus thrombosis (rare). All require neurosurgical consultation; reconstruction is deferred until infection is controlled. See Table 5.

S5.4. Surgical Approaches and Technology — Detail

S5.4.1. Approach Selection

Surgical strategy requires careful selection of operative approach (endaural versus retroauricular) and mastoidectomy technique (CWU versus CWD). This decision depends on: underlying pathology; extent of locoregional disease; urgency; available technologies; reconstructive materials; and surgeon's experience [8,9,30].

S5.4.2. Endaural Approach

The endaural approach provides direct access to the tympanic cavity and epitympanum without retroauricular incision. Indications: atticotomy and aticoantrostomy for limited epitympanic cholesteatoma not extending into the mastoid antrum; ossicular chain assessment and reconstruction; resection of small glomus tympanicum tumours; myringoplasty and limited tympanoplasty.

S5.4.3. Retroauricular Approach

The retroauricular approach is the standard for all mastoid procedures, encompassing: cortical mastoidectomy (acute coalescent mastoiditis); antromastoidectomy; Modified Bondy technique (localised attic cholesteatoma); CWU mastoidectomy (requires second-look at 12–18 months); CWD mastoidectomy (eliminates hidden disease; higher long-term cavity care burden); tympanomastoid revision; and management of exo- and endocranial complications with neurosurgical cooperation.

S5.4.4. Microscope, Endoscope, and Combined Approaches

The operative microscope provides binocular stereoscopic vision and a stable platform for drilling but is inherently limited by line-of-sight optics [31,32]. Rigid endoscopes (0°, 30°, 45°) provide panoramic illuminated views of hidden spaces inaccessible to the microscope [33,34,35]. The combined M+E approach harnesses complementary strengths: microscope for mastoid drilling, endoscope for systematic 'second-look' before closure. Adjuncts include continuous facial nerve EMG, intraoperative CT/cone-beam CT, and neuronavigation.

S5.5. Closure Materials — Detailed Descriptions

S5.5.1. Classification and Selection Principles

Surgical closure materials are classified as autologous, biologically derived, or synthetic (permanent or bioabsorbable). Optimal selection integrates four determinants: contamination status; defect size and mechanical load demands; anatomical adjacency to critical structures; and donor tissue availability [5,6,36]. No material compensates for incomplete eradication of cholesteatoma matrix [37].

S5.5.2. Autologous Materials

Temporalis fascia: Most widely used autologous material. Thin, pliable matrix conforms to irregular defect margins. Host integration >95%; infection rate <10% in contaminated fields. Gold standard for small tegmen defects (<5 mm) and facial nerve-adjacent closures. Contraindicated for direct labyrinthine fistula repair due to fibroblastic ingrowth risk; perichondrium is correct for perilymph interface.

Cartilage with perichondrium: Structural rigidity without cortical bone bulk. Ideal for labyrinthine fistula repair, tegmen augmentation, canal reconstruction. Functional preservation 90–95% at two years. Fixed impedance may alter sound transmission near ossicular chain.

Temporalis muscle flap: Vascularised coverage for extensive erosions, cavity obliteration (CWD), posterior canal reconstruction. Suprafascial dissection reduces atrophy (36% vs 72%). Advantageous in lateral sinus repairs.

Cortical and cancellous bone grafts: Most mechanically robust for load-bearing reconstruction. Cortical chips resist pulsatile CSF. Cancellous bone provides rapid vascular ingrowth in contaminated fields. ICBG provides composite structural and biological incorporation [41,42].

Autologous fat: Easy harvest; rapidly vascularised; low infection risk. Suitable for petrous apex obliteration and small CSF fistulas. Variable resorption; no structural durability.

S5.5.3. Biologically Derived Materials

Acellular dermal matrices (ADMs): Alloderm, Dermagraft TC, Dermalogen, etc. Bioscaffolds gradually repopulated by host fibroblasts and vasculature [44,45]. Principal role: dural substitutes in tegmen and skull base repair when autologous volume is insufficient.

Collagen-based dural substitutes: DuraGen, Lyoplant, Duramatrix, TachoComb, TachoSil, etc. [44,46]. High biocompatibility; diverse mechanical configurations. TachoComb/TachoSil provide dual haemostatic and sealing function. Layered combinations with autologous fascia optimised for complex skull base defects.

S5.5.4. Synthetic Materials

Titanium mesh: Greatest mechanical rigidity. Principal indication: tegmen defects >5 mm and lateral sinus exposure. Standard technique: contour mesh, overlay with autologous fascia/cartilage to create biologic interface [47,48]. Mechanical stability >5 years; recurrence <15% in sterile fields; 40–50% in contaminated environments without biologic separation.

PTFE: Chemical inertness; resistance to biofilm adherence; excellent shapeability [6,36]. Sterile fields only. Discouraged over labyrinthine structures due to impedance mismatch. Role: external buttress beneath biologic layers in large sterile defects.

Hydroxyapatite cement: Osteoconductive rigid contouring [49]. Absolute requirement for dry sterile field during polymerisation. Contraindicated in active CSF leakage, contaminated fields, suspected osteomyelitis, and skull base applications unless complete sterility is assured. Once set, not amenable to surgical debridement.

Bioabsorbable polymers (PLA, PGA): Temporary mechanical support gradually replaced by host tissue [50]. Principal advantage: elimination of long-term foreign-body load. Critical limitation: premature degradation in high-pressure CSF. Best suited for moderate sterile tegmen defects and facial nerve-adjacent repairs.

Tissue adhesives and haemostatics: Tisseel, fibrin glue — adjunctive seals only; no structural support [51]. Surgicel, Gelfoam, TachoComb — intraoperative haemostasis [52]. BoneWax — immediate mechanical haemostasis; non-resorbable; impairs bone healing; reserved for situations where alternatives are inadequate.
