# Supplementary material for: Surgical Techniques and Materials Used in the Treatment of Complicated Otomastoiditis: A Systematic Review
Source: J Clin Med. 2026 May 19;15(10):3911. doi: 10.3390/jcm15103911 (PMC13207162; doi:10.3390/jcm15103911)
Supplement: Supplementary file 1 [file jcm-15-03911-s001.zip › PRISMA_2020_Checklist_Table S1.pdf]

SUPPLEMENTARY MATERIAL S1

PRISMA 2020 Checklist

Based on: Page MJ, McKenzie JE, Bossuyt PM, et al. The PRISMA 2020 statement: an updated guideline for reporting systematic reviews. **BMJ** 2021;372:n71. doi:10.1136/bmj.n71

For more information: http://www.prisma-statement.org/

**Manuscript title**	Surgical Techniques and Materials Used in the Treatment of Complicated Otomastoiditis: A Systematic Review
**Authors**	Zica MD, Voiosu C, Rusescu A, Ionita I, Gherasie LM, Alius OR, Bizdu Branovici A, Hăinărosie R, Zainea V
**Journal**	Journal of Clinical Medicine (JCM) — MDPI | Corresponding author: catalina.pietrosanu@umfcd.ro
**PROSPERO**	CRD42025–1370406 | Registered after manuscript submission, prior to peer review (approval pending) | Protocol elements defined a priori and unchanged

☑ Yes — fully addressed in this manuscript	⚠ Partial — addressed but could be expanded	✗ Not met — action required

Section	Item	PRISMA 2020 Checklist Item	Location in Final Manuscript	Status
**TITLE**				
Title	**1**	Identify the report as a systematic review.	Title page: “Surgical Techniques and Materials Used in the Treatment of Complicated Otomastoiditis: A Systematic Review”. The words “A Systematic Review” appear explicitly in the title.	☑ Yes — fully met
**ABSTRACT**				
Abstract	**2**	See the PRISMA 2020 for Abstracts checklist. Should include: background, objectives, eligibility criteria, information sources, methods (including risk of bias), results, limitations, conclusions, funding, registration number.	Abstract contains: Background and Objectives; Materials and Methods (“PRISMA 2020-compliant search”; databases; n=56 studies; n=4,218 patients; primary outcomes listed); PROSPERO number CRD42025–1370406; Results (key pooled estimates: OR 0.56, 91.6%, <10%); Conclusions; Limitations sentence (“Limitations include the predominance of observational studies and the absence of prospective registration prior to data extraction”).	☑ Yes — fully met
**INTRODUCTION**				

Section	Item	PRISMA 2020 Checklist Item	Location in Final Manuscript	Status
Introduction	**3**	Rationale: Describe the rationale for the review in the context of existing knowledge.	Section 1 (Introduction), paragraph 4: “the available literature on optimal material selection in complicated otomastoiditis remains fragmented and heterogeneous. The present systematic review and meta-analysis seeks to address this gap…”	☑ **Yes — fully met**
Introduction	**4**	Objectives: Provide an explicit statement of the objective(s) or question(s) the review addresses.	Section 1, final paragraph: six explicit clinical scenarios listed as review objectives (cholesteatoma bone destruction; tegmen/CSF fistulas; labyrinthine/SCC fistulas; facial nerve defects; lateral sinus/posterior fossa; carotid/jugular bulb).	☑ **Yes — fully met**
**METHODS**				
Methods	**5**	Eligibility criteria: Specify inclusion and exclusion criteria for the review and how studies were grouped for syntheses.	Section 3.3 (Inclusion and Exclusion Criteria): Four inclusion criteria listed as bullet points (surgery for middle ear/mastoid disease; closure material outcomes; ≥5 patients; ≥12-month follow-up with ≥1 primary outcome). Exclusion criteria stated (abstract-only; <5-patient case reports; non-English/non-Romanian).	☑ **Yes — fully met**
Methods	**6**	Information sources: Specify all databases, registers, websites, organisations, reference lists and other sources searched. Specify date each source was last searched.	Section 3.1 (Search Strategy): PubMed/MEDLINE, Cochrane CENTRAL, Embase, Scopus. Search period: January 2000 to December 2024. Grey literature, conference proceedings, and reference lists additionally screened.	☑ **Yes — fully met**
Methods	**7**	Search strategy: Present the full search strategies for all databases, registers and websites, including any filters applied.	Section 3.1: Full Boolean search string provided: (‘cholesteatoma’ OR ‘otomastoiditis’ OR ‘temporal bone’ OR ‘mastoidectomy’) AND (‘fistula’ OR ‘dehiscence’ OR ‘CSF leak’ OR ‘labyrinthine fistula’) AND (‘surgical material’ OR ‘fascia’ OR ‘cartilage’ OR ‘titanium mesh’ OR ‘PTFE’ OR ‘hydroxyapatite’ OR ‘bioabsorbable’). Applied to all four databases.	☑ **Yes — fully met**
Methods	**8**	Selection process: Specify methods used to decide whether a study met inclusion criteria, including number of reviewers who screened each record, whether they worked independently, and if applicable, details of automation tools used.	Section 3.2 (Study Selection): “Titles and abstracts… screened independently by two reviewers (M.D.Z. and A.R.). Full texts… assessed independently by the same two reviewers… Disagreements… resolved by consensus discussion or… by referral to a third reviewer (C.V.). No automation tools were used.”	☑ **Yes — fully met**

Section	Item	PRISMA 2020 Checklist Item	Location in Final Manuscript	Status
Methods	**9**	Data collection process: Specify methods used to collect data from reports, including number of reviewers, whether they worked independently, any processes for obtaining or confirming data, and if applicable, details of automation tools used.	Section 3.4 (Data Extraction): “Data were extracted independently by two reviewers (M.D.Z. and L.M.G.) using a standardised pre-specified data extraction form. The following variables were extracted… [full variable list]. Discrepancies… resolved by discussion; where consensus could not be reached, a third reviewer (C.V.) was consulted. No automated extraction tools were used.”	☑ **Yes — fully met**
Methods	**10**	Data items: List and define all outcomes for which data were sought. Specify whether all results compatible with each outcome domain in each study were sought.	Section 3.4 (Data Extraction), second paragraph: Primary outcomes: (1) surgical site infection rate; (2) fistula recurrence at minimum two-year follow-up; (3) two-year closure integrity rate. Secondary outcomes: (4) sensorineural hearing preservation rate; (5) facial nerve function (House-Brackmann grade I–II at 12 months); (6) vestibular function (absence of persistent postoperative vertigo). “All results compatible with these outcome domains were extracted from each eligible study.”	☑ **Yes — fully met**
Methods	**11**	Study risk of bias assessment: Specify the methods used to assess risk of bias in the included studies, including details of the tool(s) used, how many reviewers assessed each study, and whether they worked independently.	Section 3.5 (Risk of Bias Assessment): Newcastle-Ottawa Scale (NOS) [16] for observational studies (3 domains); Cochrane RoB 2.0 [16] for 4 RCTs (5 domains). “All assessments were performed independently by two reviewers; disagreements were resolved by consensus.”	☑ **Yes — fully met**
Methods	**12**	Effect measures: Specify for each outcome the effect measure(s) (e.g., risk ratio, mean difference) used in the synthesis or presentation of results.	Section 3.7 (Statistical Analysis): “For binary outcomes, the effect measure was the odds ratio (OR) with 95% confidence interval (CI). Proportional outcomes (infection rate, closure integrity) were pooled as event rates with 95% CI.”	☑ **Yes — fully met**
Methods	**13**	Synthesis methods: Describe the processes used to decide which studies were eligible for each synthesis; describe any methods required to prepare data; describe statistical synthesis methods; describe any methods to explore heterogeneity.	Section 3.7 (Statistical Analysis): DerSimonian–Laird random-effects model for ≥3 studies per outcome; I^2 thresholds defined (<40% low, 40–60% moderate, >60% high); three pre-specified subgroup analyses (contamination grade; material category; defect size); Review Manager 5.4.	☑ **Yes — fully met**
Methods	**14**	Reporting bias assessment: Describe any methods used to assess risk of bias due to missing results in a synthesis (arising from reporting biases).	Section 3.6 (Reporting Bias Assessment): “For primary outcomes with ten or more contributing studies, potential publication bias was assessed by visual inspection of funnel plots and by Egger’s	☑ **Yes — fully met**

Section	Item	PRISMA 2020 Checklist Item	Location in Final Manuscript	Status
			regression test." Acknowledged as potential limitation.	
Methods	**15**	Certainty assessment: Describe any methods used to assess certainty (or confidence) in the body of evidence for an outcome.	Section 3.5 (Risk of Bias Assessment), final sentence: "The overall certainty of evidence was assessed using the GRADE approach [16]." Results reported in Discussion and Supplementary Table S4.	☑ **Yes — fully met**
**RESULTS**				
Results	**16**	Study selection: Describe the results of the search and selection process, including findings of the search of other sources, and report the number of studies included, using a flow diagram.	Section 3.2: PRISMA 2020 Flow Diagram present as Figure 1. Abstract states 56 eligible studies (n=4,218 patients) selected after systematic screening.	☑ **Yes — fully met**
Results	**17**	Study characteristics: Cite each included study and present its characteristics.	Section 8.1: "Characteristics of all 56 included studies are presented in Supplementary Table S2." Supplementary Table S2 contains: first author, year, country, study design, n patients, follow-up, population, intervention/material, outcomes, country.	☑ **Yes — fully met**
Results	**18**	Risk of bias in studies: Present assessments of risk of bias for each included study.	Section 8.1: "Risk of bias assessments for all included studies are presented in Supplementary Table S3. Overall, the majority of included studies were rated as moderate risk of bias, primarily due to their retrospective design and absence of control groups."	☑ **Yes — fully met**
Results	**19**	Results of individual studies: For all outcomes, present, for each study, (a) summary statistics for each group (where appropriate) and (b) an effect estimate and its precision, ideally using structured tables or plots.	Section 8: Figure 2 (forest plot) presents per-study data for infection rate. Tables 1–4 summarise outcomes by defect type and material. Pooled estimates reported per outcome in Sections 8.1–8.3. Supplementary Tables S2–S3 provide additional per-study data.	⚠ **Partial**
Results	**20**	Results of syntheses: For each synthesis, briefly summarise the characteristics and risk of bias among contributing studies. Report the results of all statistical syntheses performed. If meta-analysis was done, report for each: summary estimate and its precision, and measures of statistical heterogeneity.	Section 8.1: Pooled infection rates with 95% CI for autologous (7.2%, 5.1–9.8%), synthetic (18.4%, 14.2–23.1%), hybrid (9.8%, 7.2–13.0%); I^2=38% and 42% stated. Section 8.2: OR 3.8 (95% CI 1.9–7.6; p<0.001). Section 8.3: OR 5.2 (95% CI 2.8–9.6). Figure 2 (forest plot) present. Supplementary Table S4 (GRADE summary) referenced.	☑ **Yes — fully met**

Section	Item	PRISMA 2020 Checklist Item	Location in Final Manuscript	Status
Results	**21**	Reporting biases: Present assessments of risk of bias due to missing results (arising from reporting biases) for each synthesis assessed.	Section 8.1: “Visual inspection of funnel plots for the primary outcome of infection rate (k = 24 studies) demonstrated broadly symmetrical distribution. Egger’s test was not statistically significant (p = 0.18), suggesting no evidence of substantial publication bias for this outcome.” Caveat regarding retrospective studies included.	☑ **Yes — fully met**
Results	**22**	Certainty of evidence: Present assessments of certainty (or confidence) in the body of evidence for each outcome assessed.	Section 8.1: Supplementary Table S4 referenced: “A GRADE summary of findings for all primary and secondary outcomes is presented in Supplementary Table S4.” Discussion states: “According to GRADE, the certainty of evidence for the primary outcomes is moderate, with downgrading mainly due to risk of bias and inconsistency.”	☑ **Yes — fully met**
**DISCUSSION**				
Discussion	**23**	Discussion: Provide a general interpretation of the results in the context of other evidence. Discuss any limitations of the included evidence and of the review processes used. Discuss implications of the results for practice, policy, and future research.	Section 10 (Discussion): General interpretation present across 4 substantive paragraphs. Limitations paragraph: retrospective predominance; only 4 RCTs; GRADE moderate; PROSPERO post-submission registration acknowledged; future RCTs needed. Section 9 (Algorithmic Decision Framework): practice implications. Conclusions section: 6 numbered evidence-based conclusions.	☑ **Yes — fully met**
**OTHER INFORMATION**				
Other	**24**	Registration and protocol: Provide registration information for the review, including register name and registration number, or state that the review was not registered. Indicate whether a review protocol exists, and if so, where it can be accessed.	Three locations in manuscript: (1) Abstract Methods: “The review was registered in PROSPERO (CRD42025–1370406).” (2) Section 3.1: “This systematic review was registered in PROSPERO (International Prospective Register of Systematic Reviews) after manuscript submission and prior to peer review, under registration number CRD42025–1370406 (approval pending).” (3) Discussion Limitations: “Additionally, this review was registered in PROSPERO (CRD42025–1370406) after manuscript submission rather than prospectively prior to data extraction; however, the review question, eligibility criteria, and outcomes were defined a priori and remained unchanged throughout, minimising the risk of outcome-reporting bias.”	☑ **Yes — fully met**

Section	Item	PRISMA 2020 Checklist Item	Location in Final Manuscript	Status
Other	**25**	Support: Describe sources of financial or other support for the review, and the role of the funders or sponsors in the review.	Funding section: "This research was supported by the 'Publish not Perish 2026' program of Carol Davila University of Medicine and Pharmacy, Bucharest, Romania. The funder had no role in the design of the study, data collection, analysis, interpretation of results, or in the decision to publish."	☑ **Yes — fully met**
Other	**26**	Competing interests: Declare any competing interests of review authors.	Conflicts of Interest section: "The authors declare no conflicts of interest."	☑ **Yes — fully met**
Other	**27**	Availability of data, code, and other materials: Report which of the following are publicly available and where they can be found: template data collection forms; data extracted from included studies; data used for all analyses; analytic code; any other materials used in the review.	Data Availability Statement: "This study is a systematic review based on previously published literature. No new primary data were generated. The standardised data extraction form and the full dataset of extracted study-level data are available from the corresponding author (catalina.pietrosanu@umfcd.ro) upon reasonable request. Statistical analyses were performed using Review Manager version 5.4 (Cochrane Collaboration, London, UK)."	☑ **Yes — fully met**

Compliance Summary

Status	Items	Out of 27
☑ Yes	**26**	27
⚠ Partial	**1**	27
✗ No	**0**	27

Note on Item 19 (Partial):
Item 19 requires per-study data for each outcome. The present manuscript addresses this through: Figure 2 (forest plot for infection rate); Tables 1–4 (stratified outcomes); and Supplementary Tables S2–S3 (study characteristics and risk of bias). To fully satisfy this item for all outcomes, forest plots or per-study results tables for secondary outcomes (hearing preservation, facial nerve function, vestibular function, recurrence) could be added as additional supplementary figures. This is recommended if the manuscript undergoes peer review revision but is not a mandatory requirement for initial submission.

Checklist completed by the authors. All location references verified against the final manuscript version submitted to JCM. PROSPERO registration CRD42025–1370406 pending approval; manuscript will be updated with the confirmed registration number once approval is received.
