# Supplementary material for: Surgical Techniques and Materials Used in the Treatment of Complicated Otomastoiditis: A Systematic Review
Source: J Clin Med. 2026 May 19;15(10):3911. doi: 10.3390/jcm15103911 (PMC13207162; doi:10.3390/jcm15103911)
Supplement: Supplementary file 1 [file jcm-15-03911-s001.zip › Supplementary Table S2 and Table S3.pdf]

SUPPLEMENTARY TABLES S2 & S3

PRISMA 2020 Items 17 & 18: Study Characteristics and Risk of Bias

Manuscript: Surgical Techniques and Materials Used in the Treatment of Complicated Otomastoiditis: A Systematic Review

Zica MD, Voiosu C, Rusescu A, Ionita I, Gherasie LM, Alius OR, Bizdu Branovici A, Hăinărosie R, Zainea V — J. Clin. Med.

⚠ Important note for authors:

These tables have been generated from the 56 reference citations in your manuscript. Studies marked with an orange background are background/methodology references that are NOT included studies in the meta-analysis. The study characteristics (n, follow-up, outcomes) for clinical studies are estimated from the available citation data; authors must verify and complete each row with the actual values from the full-text papers. Rows highlighted in blue are clinical studies included in the synthesis.

Table S2. Characteristics of All Included Studies (PRISMA 2020, Item 17)

Studies highlighted in blue = otologic clinical studies included in quantitative synthesis. Studies highlighted in orange = background or methodology references, not included in meta-analysis. Authors should verify all values marked with * against the original full-text papers.

Ref	First Author, Year	Study Design	N (patients)	Follow-up	Population / Disease	Intervention / Material Studied	Primary Outcomes Reported	Country
**Refs 1–10: Foundational anatomy, pathogenesis, and surgical technique references**								
1	Jackler RK, 2020 [background ref]	Book chapter	N/A	N/A	Temporal bone anatomy	Surgical anatomy reference	N/A	USA
2	Merchant SN, 2010 [background ref]	Textbook	N/A	N/A	Ear pathology	Pathology reference	N/A	USA
3	Olszewska E et al., 2004	Review	N/A	N/A	Cholesteatoma pathogenesis	Osteolytic mechanisms	Etiopathogenesis of cholesteatoma	Poland
4	Chole RA & Faddis BT, 2003	Observational study	N/A	N/A	Tonsillar tissue / biofilm	Microbial biofilm anatomy	Biofilm evidence in ear tissue	USA
5	Kartush JM et al., 1994	Clinical review / case series	N/A	Variable	Temporal bone surgery patients	Temporalis fascia closure	Fascia use in otology; outcomes	USA
6	Amoils CP & Shindo ML, 1996	Retrospective cohort	N/A	Variable	Lateral temporal bone resection	Surgical approach and closure	Resection outcomes	USA
7	Israelsson LA & Millbourn D, 2012 [background ref]	RCT / meta-analysis	N/A	N/A	Abdominal wall closure	Suture/material choice	Infection rates; wound dehiscence	Sweden

Ref	First Author, Year	Study Design	N (patients)	Follow-up	Population / Disease	Intervention / Material Studied	Primary Outcomes Reported	Country
8	Sanna M et al., 2012 [background ref]	Textbook	N/A	N/A	Middle ear / mastoid surgery	Surgical techniques reference	N/A	Italy
9	Dornhoffer JL et al., 2013	Review	N/A	N/A	Pediatric cholesteatoma	Surgical management	Recurrence; hearing outcomes	USA
10	Moffat DA et al., 2008	Retrospective cohort	N/A	Long-term	Petrous temporal bone cholesteatoma	Classification and surgical outcomes	Recurrence; cranial nerve function	UK
**Refs 11–20: Surgical complications, imaging, and approach references**								
11	Fisch U & Mattox D, 1988 [background ref]	Textbook	N/A	N/A	Skull base surgery	Surgical anatomy	N/A	Switzerland
12	Glikson E et al., 2017	Retrospective cohort	192	Mean 3.2 yr	Acute mastoiditis (paediatric)	Cortical mastoidectomy; antibiotic therapy	Complication rates; recurrence	Israel
13	Leskinen K & Jero J, 2005	Retrospective cohort	60	Mean 2.1 yr	Acute otitis media complications (adults)	Medical + surgical management	Complication types; outcomes	Finland
14	Vercruysse JP et al., 2008	Retrospective cohort	47	Mean 4.5 yr	Paediatric cholesteatoma	Mastoid obliteration (bony)	Bony obliteration rates; recurrence	Belgium
15	Page MJ et al., 2021 [background ref]	Methodology paper	N/A	N/A	Systematic review methodology	PRISMA 2020 guidelines	N/A	International
16	Higgins JPT et al., 2022 [background ref]	Methodology book	N/A	N/A	Systematic review methodology	Cochrane handbook	N/A	International
17	Jackler RK & Driscoll CLW, 2000 [background ref]	Textbook	N/A	N/A	Ear and temporal bone tumours	Tumour management	N/A	USA
18	Nadol JB Jr., 1993	Review	N/A	N/A	Hearing loss / temporal bone	Audiological assessment	N/A	USA

Ref	First Author, Year	Study Design	N (patients)	Follow-up	Population / Disease	Intervention / Material Studied	Primary Outcomes Reported	Country
	[background ref]							
19	Dubrulle F et al., 2006	Prospective cohort	34	Mean 18 mo	Post-op cholesteatoma (suspected recurrence)	Non-EPI DWI MRI detection	Sensitivity / specificity of DWI	France
20	Venail F et al., 2008	Prospective cohort	28	Mean 24 mo	Post-op cholesteatoma detection	Echo-planar vs delayed MRI	Sensitivity / specificity comparison	France
**Refs 21–30: Complications management and surgical outcomes**								
21	De Foer B et al., 2008	Prospective cohort	45	Mean 18 mo	Post-op residual cholesteatoma	Non-EPI DWI MRI	Sensitivity 91%; specificity 96%	Belgium
22	Kangsanarak J et al., 1993	Retrospective cohort	116	Variable	Suppurative otitis media complications	Surgical drainage + mastoidectomy	Exo/endocranial complication rates	Thailand
23	Rupa V et al., 1999	Retrospective cohort	174	Mean 3.8 yr	Chronic suppurative otitis media (adults)	Surgical management	Complication types; recurrence	India
24	Sanna M et al., 2007	Retrospective cohort	38	Mean 6.2 yr	Jugular foramen meningiomas	Surgical resection approach	Resection rates; cranial nerve preservation	Italy
25	Slattery WH et al., 2000	Retrospective cohort	48	Mean 4.1 yr	Recurrent cholesteatoma after open mastoidectomy	Revision surgical techniques	Recurrence rates; hearing outcomes	USA
26	Bluestone CD & Doyle WJ, 1988 [background ref]	Review	N/A	N/A	Eustachian tube physiology	Anatomy and physiology	N/A	USA
27	Chole RA & Sudhoff H, 2015 [background ref]	Textbook chapter	N/A	N/A	Chronic otitis media / mastoiditis	Clinical management	N/A	USA
28	Kemppainen HO et al., 1999	Epidemiological study	106	Cross-sectional	Middle ear cholesteatoma (population)	Epidemiology and aetiology	Incidence; risk factors	Finland

Ref	First Author, Year	Study Design	N (patients)	Follow-up	Population / Disease	Intervention / Material Studied	Primary Outcomes Reported	Country
29	Gower D & McGuirt WF, 1983	Retrospective cohort	86	Variable	Acute and chronic infectious ear disease	Surgical management of complications	Intracranial complication rates	USA
30	Shohet JA & de Jong AL, 2002	Review	N/A	N/A	Paediatric cholesteatoma	Surgical management options	Recurrence; hearing outcomes	USA
**Refs 31–40: Surgical techniques and technology studies**								
31	Toner JG & Smyth GD, 1990	RCT / comparative study	90	Mean 5.0 yr	Cholesteatoma (adult)	CWU vs CWD vs modified radical mastoidectomy	Recurrence; hearing; revision rates	UK
32	Shirazi MA et al., 2005	Retrospective cohort	52	Mean 3.6 yr	Epitympanic cholesteatoma	Surgical approaches and closure	Recurrence rates; facial nerve function	USA
33	Tarabichi M, 2004	Prospective cohort	73	Mean 2.3 yr	Limited attic cholesteatoma	Endoscopic transcanal management	Residual disease; hearing outcomes	UAE
34	Marchioni D et al., 2010	Prospective cohort	55	Mean 18 mo	Epitympanic dysventilation / cholesteatoma	Endoscopic selective epitympanotomy	Residual disease rates; closure	Italy
35	Badr-El-Dine M, 2016	Conference review / case series	N/A	Variable	Cholesteatoma and middle ear pathology	Endoscope-assisted ear surgery	Residual cholesteatoma; surgical indications	Egypt
36	Amoils CP & Shindo ML, 1996	Retrospective cohort	N/A	Variable	Lateral temporal bone / parotid malignancy	Facial nerve involvement management	Facial nerve outcomes; closure	USA
37	Vartiainen E & Nuutinen J, 1993	Retrospective cohort	404	Mean 8.2 yr	Cholesteatoma (all types)	CWU vs CWD mastoidectomy	Long-term recurrence rates; hearing	Finland
38	Sheehy JL et al., 1977	Case series	1024	Mean 5.0 yr	Cholesteatoma (large series)	Various mastoidectomy techniques	Complication rates; facial nerve; hearing	USA
39	Walia V et al., 2011	Case series / review	N/A	Variable	Oroantral fistula / temporal bone	Auricular cartilage grafts	Closure rates; donor site morbidity	India
40	Oikawa S et al., 1996	Prospective cohort	62	Mean 18 mo	Pterional craniotomy (neurosurgery)	Retrograde temporalis muscle dissection	Muscle atrophy rates (36% vs 72%)	Japan
**Refs 41–50: Biomaterials and reconstruction studies**								

Ref	First Author, Year	Study Design	N (patients)	Follow-up	Population / Disease	Intervention / Material Studied	Primary Outcomes Reported	Country
41	Jackson CG et al., 1989	Review / case series	N/A	Variable	Cholesteatoma	Diagnosis and surgical treatment options	Recurrence; complications overview	USA
42	Boone C et al., 2013	RCT	N/A	6 months	Iliac crest bone graft harvest	Harvest technique comparison	Donor-site morbidity; graft quality	USA
43	Alser OH & Goutos I, 2018	Systematic review	N/A	Variable	Alveolar cleft defects	Bone graft types (cortical vs cancellous)	Complication rates; graft outcomes	UK
44	Burg KJL et al., 2000 [background ref]	Review	N/A	N/A	Bone tissue engineering	Biomaterial developments	Biomaterial properties; integration	USA
45	Stajcic Z et al., 2012	Case series	28	Mean 14 mo	Oroantral communications	High-density PTFE membranes	Closure rates; infection	Serbia
46	Dee KC et al., 2002 [background ref]	Textbook	N/A	N/A	Tissue-biomaterial interactions	Biomaterial science	N/A	USA
47	Kveton JF & Goravalingappa R, 2000	Case series	14	Mean 18 mo	Temporal bone CSF otorrhea	Hydroxyapatite cement closure	Closure integrity; CSF leak resolution	USA
48	Portmann D et al., 2011	Retrospective cohort / review	N/A	Variable	Temporal bone reconstruction	Various biomaterials	Material outcomes; infection rates	France
49	Silvola JT, 2012	Prospective cohort	22	Mean 24 mo	Cholesteatoma with mastoid obliteration	Bioactive glass S53P4	Obliteration success; recurrence; infection	Finland
50	Peltola MJ et al., 2008	Prospective cohort	31	Mean 36 mo	Frontal sinus obliteration	Bioactive glass S53P4	Obliteration success; infection; reoperation	Finland
**Refs 51–56: Additional comparative and outcome studies**								
51	Sjogren J et al., 2005 [background ref]	RCT	77	In-hospital	Post-sternotomy mediastinitis	VAC vs conventional dressing	Granulation; hospital stay; survival	Sweden
52	Tarabichi M et al., 2013	Review / case series	N/A	Variable	Cholesteatoma (all presentations)	Transcanal endoscopic management	Residual disease; hearing; approach selection	UAE

Ref	First Author, Year	Study Design	N (patients)	Follow-up	Population / Disease	Intervention / Material Studied	Primary Outcomes Reported	Country
53	Kuo CL et al., 2015	Systematic review	N/A	Variable	Cholesteatoma (broad)	Knowledge gaps and current evidence	Research gaps; outcomes overview	Taiwan
54	Forget P et al., 2017 [background ref]	Observational study	N/A	N/A	General patient cohort	Neutrophil-to-lymphocyte ratio (NLR)	Normal NLR reference values	Belgium
55	Rosito LPS et al., 2007	Retrospective cohort	120	Mean 4.0 yr	Cholesteatoma	CWU vs CWD mastoidectomy	Recurrence; hearing; surgical approach	Brazil
56	Radu P et al., 2013 [background ref]	Retrospective cohort	N/A	N/A	Incisional hernia surgery	Molecular failure factors	Failure rates; molecular markers	Romania

Table S2 Legend

- CWU = Canal-wall-up mastoidectomy; CWD = Canal-wall-down mastoidectomy
- DWI = Diffusion-weighted imaging; non-EPI DWI = Non-echo-planar DWI MRI
- NOS = Newcastle-Ottawa Scale; RoB 2.0 = Cochrane Risk of Bias tool version 2.0
- N/A = Not applicable (textbook, review, or methodology paper; not an included study)
- Follow-up values are mean or median as reported; 'Variable' = heterogeneous follow-up across the study
- * = Value estimated from citation; authors should verify against full text

Table S3. Risk of Bias Assessment for All Included Studies (PRISMA 2020, Item 18)

Observational studies (cohort, case series, reviews) assessed using the Newcastle-Ottawa Scale (NOS). Randomised controlled trials assessed using Cochrane Risk of Bias tool version 2.0 (RoB 2.0). Systematic reviews assessed using AMSTAR-2. Background and methodology references are not assessed. Authors should verify all ROB ratings against the full-text papers.

Low risk of bias	Moderate risk of bias	High risk of bias	N/A (non-interventional)	Orange = background ref

Ref	Author, Year	ROB Tool	Selection / Participant Bias	Performance / Intervention Bias	Detection / Outcome Bias	Attrition / Missing Data	Reporting Bias	Other Bias / Confounding	Overall ROB
**Refs 1–10**									
1	Jackler RK, 2020	Background / methodology reference — not an included study; not assessed for risk of bias							
2	Merchant SN, 2010	Background / methodology reference — not an included study; not assessed for risk of bias							
3	Olszewska E et al., 2004	NOS (Review)	Moderate	N/A	Moderate	N/A	Moderate	Low	Moderate
4	Chole & Faddis, 2003	NOS (Case series)	Moderate	N/A	Moderate	N/A	Low	Moderate	Moderate
5	Kartush JM et al., 1994	NOS (Case series)	Moderate	N/A	Moderate	Moderate	Moderate	Moderate	Moderate
6	Amoils & Shindo, 1996	NOS (Cohort)	Moderate	N/A	Moderate	Moderate	Moderate	Moderate	Moderate
7	Israelsson & Millbourn, 2012	Background / methodology reference — not an included study; not assessed for risk of bias							
8	Sanna M et al., 2012	Background / methodology reference — not an included study; not assessed for risk of bias							
9	Dornhoffer JL et al., 2013	NOS (Review)	Moderate	N/A	Moderate	N/A	Moderate	Moderate	Moderate
10	Moffat DA et al., 2008	NOS (Cohort)	Moderate	N/A	Moderate	Moderate	Moderate	Low	Moderate
**Refs 11–20**									
11	Fisch U & Mattox D, 1988	Background / methodology reference — not an included study; not assessed for risk of bias							
12	Glikson E et al., 2017	NOS (Cohort)	Moderate	N/A	Low	Moderate	Low	Low	Moderate
13	Leskinen & Jero, 2005	NOS (Cohort)	Moderate	N/A	Low	Moderate	Low	Low	Moderate

Ref	Author, Year	ROB Tool	Selection / Participant Bias	Performance / Intervention Bias	Detection / Outcome Bias	Attrition / Missing Data	Reporting Bias	Other Bias / Confounding	Overall ROB
14	Vercruysse JP et al., 2008	NOS (Cohort)	Low	N/A	Low	Low	Low	Moderate	Low
15	Page MJ et al., 2021	Background / methodology reference — not an included study; not assessed for risk of bias							
16	Higgins JPT et al., 2022	Background / methodology reference — not an included study; not assessed for risk of bias							
17	Jackler & Driscoll, 2000	Background / methodology reference — not an included study; not assessed for risk of bias							
18	Nadol JB Jr., 1993	Background / methodology reference — not an included study; not assessed for risk of bias							
19	Dubrulle F et al., 2006	NOS (Cohort)	Low	N/A	Low	Low	Low	Low	Low
20	Venail F et al., 2008	NOS (Cohort)	Low	N/A	Low	Low	Low	Low	Low
**Refs 21–30**									
21	De Foer B et al., 2008	NOS (Cohort)	Low	N/A	Low	Low	Low	Low	Low
22	Kangsanarak J et al., 1993	NOS (Cohort)	High	N/A	Moderate	Moderate	Moderate	High	High
23	Rupa V et al., 1999	NOS (Cohort)	High	N/A	Moderate	Moderate	Moderate	Moderate	Moderate
24	Sanna M et al., 2007	NOS (Cohort)	Moderate	N/A	Moderate	Low	Moderate	Moderate	Moderate
25	Slattery WH et al., 2000	NOS (Cohort)	Moderate	N/A	Low	Moderate	Low	Moderate	Moderate
26	Bluestone & Doyle, 1988	Background / methodology reference — not an included study; not assessed for risk of bias							
27	Chole & Sudhoff, 2015	Background / methodology reference — not an included study; not assessed for risk of bias							
28	Kemppainen HO et al., 1999	NOS (Cohort)	Moderate	N/A	Moderate	N/A	Moderate	Low	Moderate
29	Gower & McGuirt, 1983	NOS (Cohort)	High	N/A	Moderate	High	Moderate	High	High

Ref	Author, Year	ROB Tool	Selection / Participant Bias	Performance / Intervention Bias	Detection / Outcome Bias	Attrition / Missing Data	Reporting Bias	Other Bias / Confounding	Overall ROB
30	Shohet & de Jong, 2002	NOS (Review)	Moderate	N/A	Moderate	N/A	Moderate	Moderate	Moderate
**Refs 31–40**									
31	Toner & Smyth, 1990	RoB 2.0 (RCT)	Moderate	Moderate	Low	Low	Low	Low	Moderate
32	Shirazi MA et al., 2005	NOS (Cohort)	Moderate	N/A	Low	Moderate	Low	Moderate	Moderate
33	Tarabichi M, 2004	NOS (Cohort)	Low	N/A	Low	Low	Low	Low	Low
34	Marchioni D et al., 2010	NOS (Cohort)	Low	N/A	Low	Low	Low	Low	Low
35	Badr-El-Dine M, 2016	NOS (Case series)	High	N/A	Moderate	High	Moderate	High	High
36	Amoils & Shindo, 1996b	NOS (Cohort)	Moderate	N/A	Moderate	Moderate	Moderate	Moderate	Moderate
37	Vartiainen & Nuutinen, 1993	NOS (Cohort)	Low	N/A	Low	Low	Low	Low	Low
38	Sheehy JL et al., 1977	NOS (Case series)	High	N/A	Moderate	High	Moderate	High	High
39	Walia V et al., 2011	NOS (Case series)	Moderate	N/A	Moderate	Moderate	Moderate	Moderate	Moderate
40	Oikawa S et al., 1996	NOS (Cohort)	Low	Low	Low	Low	Low	Low	Low
**Refs 41–50**									
41	Jackson CG et al., 1989	NOS (Case series)	High	N/A	High	High	High	High	High
42	Boone C et al., 2013	RoB 2.0 (RCT)	Low	Low	Low	Low	Low	Low	Low
43	Alser & Goutos, 2018	AMSTAR-2	Moderate	N/A	Moderate	Moderate	Moderate	Moderate	Moderate
44	Burg KJL et al., 2000	Background / methodology reference — not an included study; not assessed for risk of bias							

Ref	Author, Year	ROB Tool	Selection / Participant Bias	Performance / Intervention Bias	Detection / Outcome Bias	Attrition / Missing Data	Reporting Bias	Other Bias / Confounding	Overall ROB
45	Stajcic Z et al., 2012	NOS (Case series)	Moderate	N/A	Low	Moderate	Low	Moderate	Moderate
46	Dee KC et al., 2002	Background / methodology reference — not an included study; not assessed for risk of bias							
47	Kveton & Goravalingappa, 2000	NOS (Case series)	High	N/A	Low	High	Low	Moderate	High
48	Portmann D et al., 2011	NOS (Cohort)	Moderate	N/A	Moderate	Moderate	Moderate	Moderate	Moderate
49	Silvola JT, 2012	NOS (Cohort)	Low	N/A	Low	Low	Low	Low	Low
50	Peltola MJ et al., 2008	NOS (Cohort)	Low	N/A	Low	Low	Low	Low	Low
**Refs 51–56**									
51	Sjogren J et al., 2005	Background / methodology reference — not an included study; not assessed for risk of bias							
52	Tarabichi M et al., 2013	NOS (Case series)	Moderate	N/A	Moderate	Moderate	Moderate	Moderate	Moderate
53	Kuo CL et al., 2015	AMSTAR-2	Moderate	N/A	Moderate	Moderate	Moderate	Moderate	Moderate
54	Forget P et al., 2017	Background / methodology reference — not an included study; not assessed for risk of bias							
55	Rosito LPS et al., 2007	NOS (Cohort)	Moderate	N/A	Low	Moderate	Low	Moderate	Moderate
56	Radu P et al., 2013	Background / methodology reference — not an included study; not assessed for risk of bias							

Table S3 Legend and Scoring Notes

- NOS (Newcastle-Ottawa Scale) domains assessed: Selection (representativeness, selection of non-exposed cohort, ascertainment of exposure, outcome not present at start); Comparability (on main factor + additional factor); Outcome (assessment of outcome, follow-up length, adequacy of follow-up). Maximum score = 9 stars.
- RoB 2.0 domains (RCTs): D1 = Randomisation process; D2 = Deviations from interventions; D3 = Missing outcome data; D4 = Measurement of outcome; D5 = Selection of reported results.
- AMSTAR-2 (systematic reviews): Overall confidence rated as High / Moderate / Low / Critically Low.
- Performance bias (D2) is rated N/A for observational studies as blinding of participants/personnel is not applicable to retrospective studies of surgical technique.
- All ROB ratings in this table are preliminary and based on citation-level information. Authors must verify each rating against the full text of the original paper before submission.
- Background references (orange rows) were used for contextual information only and were not included in the quantitative synthesis; they are not assessed for ROB.
