# Supplementary material for: Surgical Techniques and Materials Used in the Treatment of Complicated Otomastoiditis: A Systematic Review
Source: J Clin Med. 2026 May 19;15(10):3911. doi: 10.3390/jcm15103911 (PMC13207162; doi:10.3390/jcm15103911)
Supplement: Supplementary file 1 [file jcm-15-03911-s001.zip › Supplementary Table S4.pdf]

Table 5. Summary of Findings: GRADE Certainty of Evidence for Primary and Secondary Outcomes

⊕⊕⊕⊕ HIGH	⊕⊕⊕⊘ MODERATE	⊕⊕⊘⊘ LOW	⊕⊘⊘⊘ VERY LOW
Further research very unlikely to change our confidence in the effect estimate	Further research likely to have important impact on our confidence in the effect estimate	Further research very likely to have important impact on our confidence in the effect estimate	We are very uncertain about the estimate

Outcome	Studies (n)	Patients (n)	Effect Estimate (95% CI)	I²	GRADE domains — reasons for downgrading certainty					GRADE Certainty
(values taken directly from manuscript Sections 8.1, 8.2, 8.3)					Risk of bias	Inconsistency (heterogeneity)	Indirectness (population/outcome)	Imprecision (CI width / n)	Publication bias	⊕ = not serious ⊘ = serious
**Primary outcomes**										
Infection rate: autologous vs. synthetic closure materials (all fistula types; all contamination grades combined)	24 a	~1,900	Autologous: 7.2% (95% CI 5.1–9.8%) Synthetic: 18.4% (95% CI 14.2–23.1%) Hybrid: 9.8% (95% CI 7.2–13.0%)	38%	Serious	Not serious	Not serious	Not serious	Undetected	Moderate ⊕⊕⊕⊘
Two-year closure integrity rate (by material category; all defect types)	18 b	~1,400	Autologous: 89.4% Synthetic: 87.1% Hybrid: 91.6% (hybrid vs. autologous RD: +2.2%)	42%	Serious	Serious	Not serious	Not serious	Undetected	Moderate ⊕⊕⊕⊘
Fistula recurrence at 5 years (residual cholesteatoma matrix as predictor)	12 c	~980	OR 5.2 (95% CI 2.8–9.6) for residual matrix vs. complete clearance	31%	Serious	Not serious	Not serious	Not serious	Undetected	Moderate ⊕⊕⊕⊘
**Secondary outcomes**										
Sensorineural hearing preservation	8 d	~420	92.3% vs. 74.6% OR 3.8	29%	Serious	Not serious	Not serious	Not serious	Undetected	Moderate ⊕⊕⊕⊘

Outcome	Studies (n)	Patients (n)	Effect Estimate (95% CI)	I²	GRADE domains — reasons for downgrading certainty					GRADE Certainty
(values taken directly from manuscript Sections 8.1, 8.2, 8.3)					Risk of bias	Inconsistency (heterogeneity)	Indirectness (population/outcome)	Imprecision (CI width / n)	Publication bias	⊕ = not serious ⊘ = serious
(labyrinthine and SCC fistula repairs; autologous cartilage-fascia vs. synthetic-dominant; 2-year follow-up)			(95% CI 1.9–7.6; p<0.001)							
Facial nerve function preservation (House-Brackmann grade I–II at 12 months; thin fascia vs. synthetic without fascial buffering)	7 e	~380	95.4% (fascia only) vs. 87.1% (synthetic without buffering)	N/A	Serious	Not serious	Not serious	Serious	Undetected	Low ⊕⊕⊘⊘
Vestibular function / absence of persistent postoperative vertigo (direct labyrinthine contact — autologous vs. rigid synthetic)	6 f	~310	4.2% vertigo (autologous) vs. 13.8% (rigid synthetic in direct labyrinthine contact)	N/A	Serious	Not serious	Not serious	Serious	Undetected	Low ⊕⊕⊘⊘
**Surgical technology outcome**										
Residual cholesteatoma at 2 years (endoscope-assisted M+E vs. microscope-alone surgery; CWU and	12 g	~860	OR 0.56 (95% CI 0.38–0.82; p=0.003) I²=41%; favours combined M+E approach	41%	Serious	Not serious	Not serious	Not serious	Undetected	Moderate ⊕⊕⊕⊘

Outcome	Studies (n)	Patients (n)	Effect Estimate (95% CI)	I²	GRADE domains — reasons for downgrading certainty					GRADE Certainty
(values taken directly from manuscript Sections 8.1, 8.2, 8.3)					Risk of bias	Inconsistency (heterogeneity)	Indirectness (population/outcome)	Imprecision (CI width / n)	Publication bias	⊕ = not serious ∅ = serious
revision procedures)										

Footnotes and basis for GRADE domain ratings:

a 24 studies contributed to the pooled infection rate analysis. Exact study count to be confirmed from Supplementary Table S2 on submission.

b 18 studies reported two-year closure integrity data; heterogeneity moderate (I^2=42%) — one downgrade for inconsistency applied.

c 12 studies reported fistula recurrence at ≥5-year follow-up; OR 5.2 (95% CI 2.8–9.6) for residual cholesteatoma matrix.

d 8 studies reported hearing preservation outcomes in labyrinthine/SCC fistula repairs (Section 8.2).

e 7 studies reported facial nerve function using House-Brackmann grading at 12 months. Imprecision rated serious due to wide confidence intervals and limited sample sizes in individual studies.

f 6 studies reported vestibular outcomes (persistent vertigo rates). Imprecision rated serious given small total patient numbers.

g 12 studies contributed to the endoscope vs. microscope comparison; OR 0.56 (95% CI 0.38–0.82); I^2=41% (Section 8 / Figure 2).

⊕ ⊕ = not serious downgrade for this domain; ∅ = serious downgrade applied.

ROB Risk of bias rated Serious for all outcomes reflecting the predominance of retrospective cohort studies and case series (only 4 RCTs included). This is the primary reason all outcomes are rated Moderate or Low, consistent with the GRADE statement in Section 10: 'the certainty of evidence for the primary outcomes is moderate, with downgrading mainly due to risk of bias and inconsistency.'

Pub Publication bias rated Undetected: funnel plot inspection was not statistically significant (Egger’s test p=0.18 for infection rate; Section 8.1).

GRADE GRADE approach applied per Guyatt GH et al. (2011); certainty assessed as: High, Moderate, Low, or Very Low. Facial nerve (Item e) and vestibular outcomes (Item f) rated Low due to additional serious imprecision from limited sample sizes.

CI = confidence interval; OR = odds ratio; RD = risk difference; SCC = semicircular canal; M+E = combined microscope-endoscope approach; CWU = canal-wall-up mastoidectomy; HB = House-Brackmann grading scale; GRADE = Grading of Recommendations Assessment, Development and Evaluation.
